# A family cluster of diagnosed coronavirus disease 2019 (COVID‐19) kidney transplant recipient in Thailand

**DOI:** 10.1002/iid3.337

**Published:** 2020-08-08

**Authors:** Parichart Sakulkonkij, Jackrapong Bruminhent, Charan Pankongngam, Nipon Chalermphunchai

**Affiliations:** ^1^ Division of Infectious Diseases, Internal Medicine Department Lampang Hospital Lampang Thailand; ^2^ Division of Infectious Diseases, Department of Medicine, Faculty of Medicine Ramathibodi Hospital Mahidol University Bangkok Thailand

**Keywords:** COVID‐19, family cluster, pneumonia, post kidney transplant, transmission

## Abstract

**Introduction:**

Severe acute respiratory syndrome coronavirus 2 (SARS‐CoV‐2) causes an ongoing outbreak of respiratory illness called coronavirus disease 2019 (COVID‐19). The clinical course could be ranging from mild to severe illness especially the individuals with an immunocompromised condition such as solid organ transplant recipients.

**Method:**

We described a family cluster of COVID‐19 patients who were admitted during 3rd April 2020 to 30th April 2020. COVID‐19 was confirmed by a presence of SARS‐CoV‐2 ribonucleic acid in the respiratory specimens detected by a qualitative, real‐time reverse transcription‐polymerase chain reaction. The study focused on the clinical course and management of our cases.

**Results:**

A family cluster of four laboratory‐confirmed COVID‐19 patients, one of those carried an underlying kidney transplant (KT) receiving immunosuppressants. Clinical presentation and severity of our case series are variable depending on each individual immune status. By far, a KT recipient seems to develop more severity despite antiviral therapy, cessation of immunosuppressant, and aggressive intensive care support.

**Conclusion:**

Our case series plausibly affirmed a person‐to‐person transmission and potentially severe disease in the transplant population. Clinicians who are encountering with transplant recipients should be aware of possible transmission among family members.

## Graphical abstract

A family cluster of four laboratory‐confirmed COVID‐19 patients. Our case series plausibly affirmed a person‐to‐person transmission and potentially severe disease in the transplant population. Clinicians who are encountering with transplant recipients should be aware of possible transmission among family members.

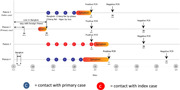

**Abbreviations**
COVID‐19: coronavirus disease 2019
CRP: C‐reactive protein
GFR: glomerular filtration rate
KT: kidney transplant
LDH: lactate dehydrogenase
RT‐PCR: reverse transcription‐polymerase chain reaction
SARS‐CoV‐2: severe acute respiratory syndrome coronavirus
SOT: solid organ transplant
WBC: white blood cell

## 1 INTRODUCTION

A novel betacoronavirus, the seventh member of coronaviruses, which is shown to infect humans and lately named as severe acute respiratory syndrome coronavirus 2 (SARS‐CoV‐2) causes an ongoing outbreak of respiratory illness that began in December 2019 in China called coronavirus disease 2019 (COVID‐19). The first COVID‐19 case in Thailand, also the first recorded case outside of China, was reported in mid‐January 2020.^1^ Up till early May 2020, approximately 3000 cases were reported in Thailand with an estimated 1.8% mortality rate.^1^ The clinical course of the disease seems to be mild in the majority especially those who are young and free of comorbidities. However, those who are elderly, occupied by chronic medical conditions, or immunocompromised tend to have more severe disease and mortality rates.^2^, ^3^, ^4^, ^5^, ^6^, ^7^, ^8^ The SARS‐CoV‐2 is mainly spread through droplets and contact thus far.^9^ Therefore, person‐to‐person transmission of this virus has been reported both in family and hospital in the literature.^6^, ^10^, ^11^, ^12^, ^13^ In general, the median incubation period was reported to be as 5 days and almost 98% would develop symptoms within 12 days of infection.^14^ The established risk factors of COVID‐19 acquisition among Thai patients were close contact with an index case or a previous journey to a high‐risk area.^15^, ^16^ Therefore, a definition of patients under investigation provided by the Ministry of Public Health of Thailand was provided and encouraged to specify those should be tested for COVID‐19. Furthermore, a lockdown policy has been implemented to prevent transmission across the country.^17^ Kidney transplant (KT) recipients were considered as the largest population among other solid organs in Thailand. Although the clinical characteristics of KT recipients who were diagnosed with COVID‐19 have been reported during the course this pandemic, a cluster of transplant recipient's family members have not been much explored.^18^ Furthermore, an outcome of KT recipients who were treated with antiviral agent, favipiravir has not been much described. Therefore, we described a cluster of COVID‐19 among four laboratory‐confirmed COVID‐19 patients in the same family in a city located in the northern part of Thailand (Table 1).

**Table 1:** Demographic, clinical, laboratory, treatment and outcome of patients with COVID‐19

Characteristics	Case 1 (index case)	Case 2 (primary case)	Case 3	Case 4
Age, y	58	42	64	38
Sex	Female	Female	Female	Male
Comorbidity	COPD	None	None	Kidney transplant
Risk factors	Sick contact	Travel history	Sick contact	Sick contact
Duration of symptoms before admission, d	3	6	0	4
Symptoms at onset of illness				
Fever	Yes	No	No	Yes
Productive cough	Yes	Yes	No	No
Sore throat	No	Yes	No	No
Myalgia	No	No	No	Yes
Laboratory finding				
Lymphopenia < 1500/mm^3^	Yes	Yes	Yes	Yes
Thrombocytopenia < 140 000	Yes	No	Yes	Yes
Disease severity	Moderate	Mild	Severe	Severe
Complication	None	None	Acute respiratory distress syndrome	Allograft dysfunction
Treatment				
Antiviral therapy	Yes	Yes	Yes	Yes
Antibiotic therapy	No	No	Yes	Yes
Use of corticosteroid	No	No	No	No
Renal replacement therapy	⋯	⋯	⋯	⋯
Oxygen support				
Nasal cannula	Yes	No	No	No
High flow oxygen cannula	No	No	No	Yes
Invasive mechanical ventilation	No	No	Yes	No
Prone position	No	No	Yes	Yes
Outcome	Survived	Survived	Survived	Survived

Abbreviations: COPD, chronic obstructive pulmonary disease; COVID‐19, coronavirus disease 2019.

## 2 CASE PRESENTATION

### 2.1 Case 1 (index case)

**Figure 1:**
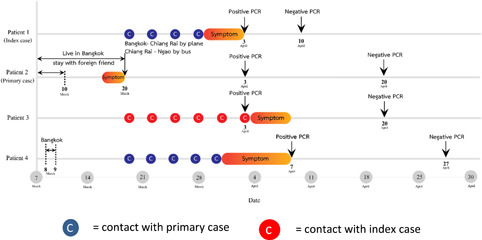
Timeline of exposure history of a family cluster of COVID‐19. COVID‐19, coronavirus disease 2019; PCR, polymerase chain reaction

**Figure 2:**
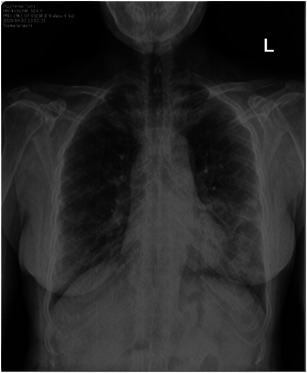
Chest X‐ray of an index case (Case 1) on the day of admission revealed new small patchy opacities in left lower lung field

A 58‐year‐old female who presented with fever and productive cough of 2 days duration on 2nd April 2020. She lived in Ngao, a city located in the northern region of Thailand. Her temperature was 37.9°C. The throat and lung exams did not reveal abnormal findings. She had chronic obstructive pulmonary disease (COPD) which was controlled by fluticasone/salmeterol and fenoterol/ipratropium bromide metered‐dose inhalers. She reported a history of contact with her daughter who had recently returned home from Bangkok, a city located in the central region of Thailand 2 weeks before this presentation. A timeline event of the cluster is shown in Figure 1. On admission, a nasopharyngeal and throat swabs for SARS‐CoV‐2 reverse transcription‐polymerase chain reaction (RT‐PCR) revealed a positive result, other laboratory findings included white blood cell count (WBC) 2480 cells/mm^3^, lymphocyte (L) 18%, neutrophil (N) 78%, and C‐reactive protein (CRP) 62.7 mg/L. The chest X‐ray (CXR) revealed new small patchy opacities in the left lower lung field as shown in Figure 2. Therefore, she was diagnosed with COVID‐19 pneumonia and treated with hydroxychloroquine 800 mg/d, ritonavir‐boosted lopinavir 800 mg/d, and favipiravir 1200 mg/d for a total of 10 days and azithromycin 500 mg/d for 5 days. Her respiratory status had been stable with nasal cannula 3 L/minute (liters per minute, LPM) support and gradually improved. A repeated nasopharyngeal and throat swabs for SARS‐CoV‐2 RT‐PCR was negative after 7 days of treatment.

### 2.2 Case 2 (primary case)

A 42‐year‐old healthy female (the daughter of Case 1) who had worked in Bangkok as a businessman. She had contacted with a foreign visitor 3 weeks before the event. Few days before visiting her mother, she had fever with productive cough and sore throat however, her symptoms were spontaneously resolved without medical attention few days later. Then she was admitted because the history of closed contact with confirmed COVID‐19 and was found to be positive polymerase chain reaction (PCR) for SARS‐CoV2 from nasopharyngeal and throat swab. The physical examination was unremarkable. Blood test showed WBC 6260 cells/mm^3^ and L 31%. Her CXR was unremarkable. Therefore, she was diagnosed with mild COVID‐19, and treated with hydroxychloroquine 1200 mg/d, ritonavir‐boosted lopinavir 800 mg/d, and roxithromycin 300 mg/d. Finally, her clinical had improved and a repeated PCR for SARS‐CoV2 from nasopharyngeal and throat swab turned to negative on day 17 after treatment.

### 2.3 Case 3

**Figure 3:**
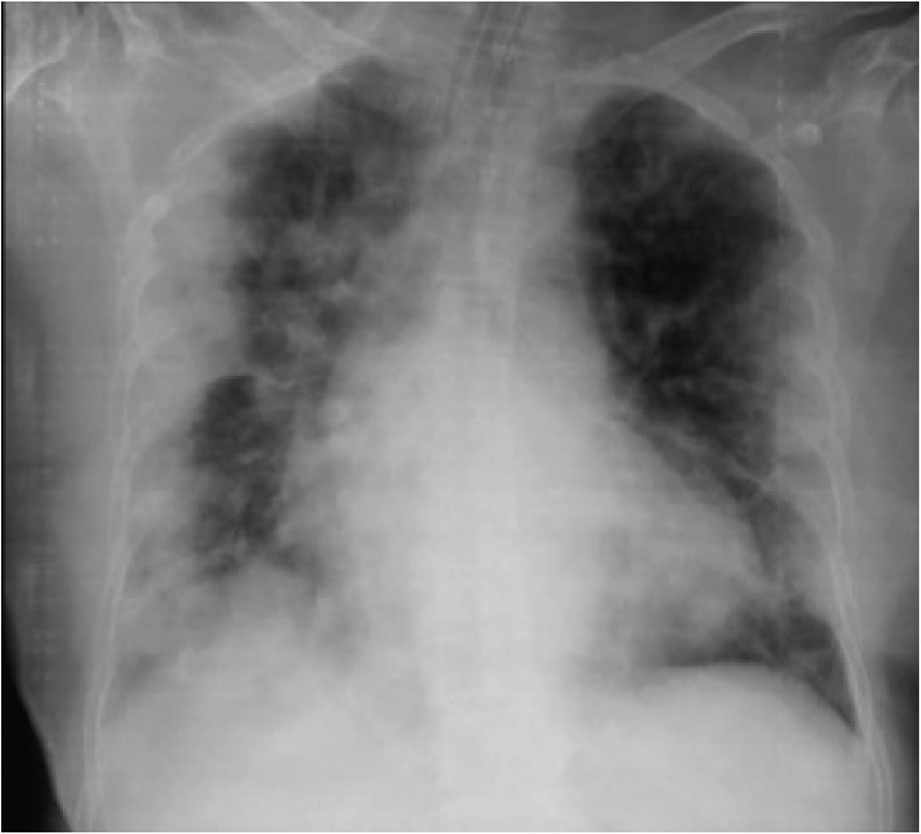
Chest X‐ray of Case 3 on day 9 after onset of illness. Patchy opacities in peripheral and basal both lungs, which was consistent with clinical acute respiratory distress syndrome. Finally, the infiltrations were all resolved at the time of recovery on day 25 after onset of illness

A 64‐year‐old healthy female (sister of Case 1) who lived nearby and reported visiting an index case every day. Although she reported being asymptomatic, her nasopharyngeal and throat swab for SARS‐CoV‐2 PCR was positive, and the CXR was unremarkable on admission. However, her respiratory symptoms had been progressing along with the CXR, which revealed the progression to patchy opacities in peripheral and basal both lungs. The diagnosis of acute respiratory distress syndrome (ARDS) was confirmed by PaO_2_/FiO_2_ ratio of 82 (Figure 3) on day 9 of admission. Her WBC was 9080 cells/mm^3^ with L 11%. She was started with hydroxychloroquine 400 mg/d, ritonavir‐boosted lopinavir 400 mg/d, and favipiravir 1200 mg/d. She was placed on invasive mechanical ventilation with prone position and antibiotic was escalated to intravenous meropenem. After 10 days of treatment, her clinical and radiographic findings were improved. Finally, the tracheal aspiration for SARS‐CoV‐2 PCR turned negative on 28 days after treatment.

### 2.4 Case 4

**Figure 4:**
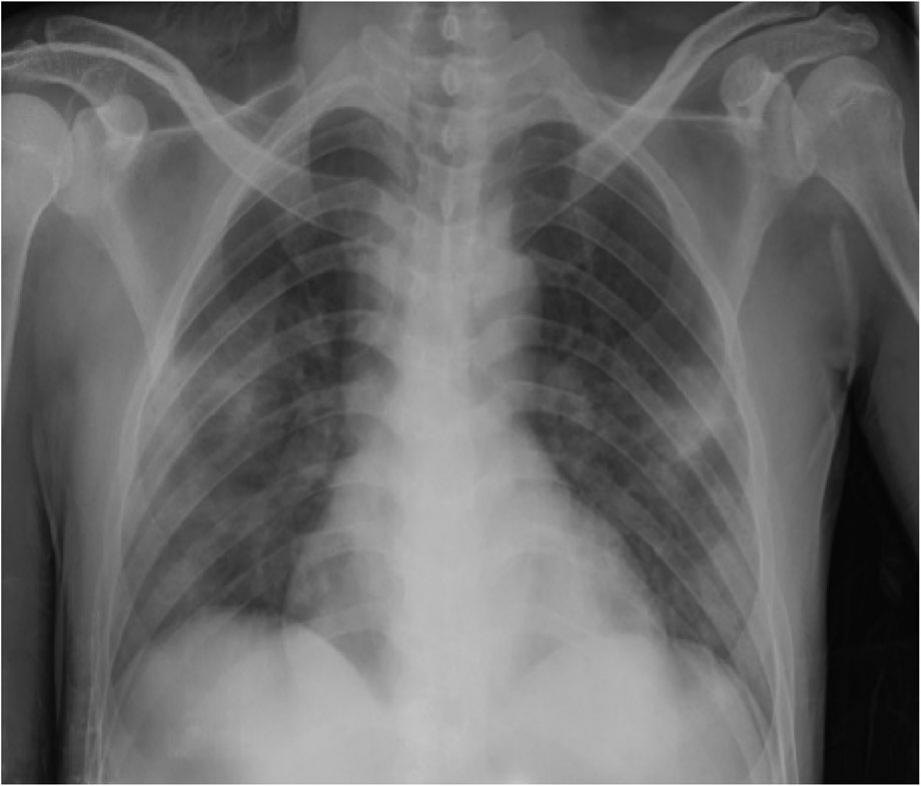
Chest X‐ray from Case 4. On days 14 after the onset of illness. Progressive of multiple small patchy opacities in peripheral and basal both lungs consistent with severe pneumonia. All the lesions were resolved on day 29 of the illness

**Figure 5:**
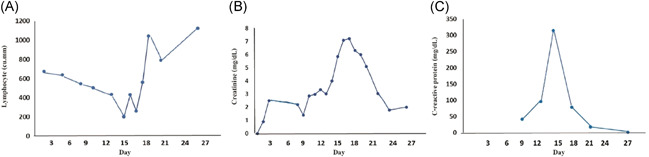
Temporal change in laboratory markers during the course of an illness in a kidney transplant recipient (Case 4) who were diagnosed with severe COVID‐19 pneumonia A, Dynamic change of lymphocyte count. Data showed progressive decline of lymphocyte count in the second week of illness these suggested as severe illness and lymphocyte count progressively increase at the recovery phase. B and C, Dynamic change of serum creatinine and CRP. Data showed increase level of serum creatinine and CRP in the second week of illness and decrease to normal at the recovery phase. COVID‐19, coronavirus disease 2019; CRP, C‐reactive protein

**Figure 6:**
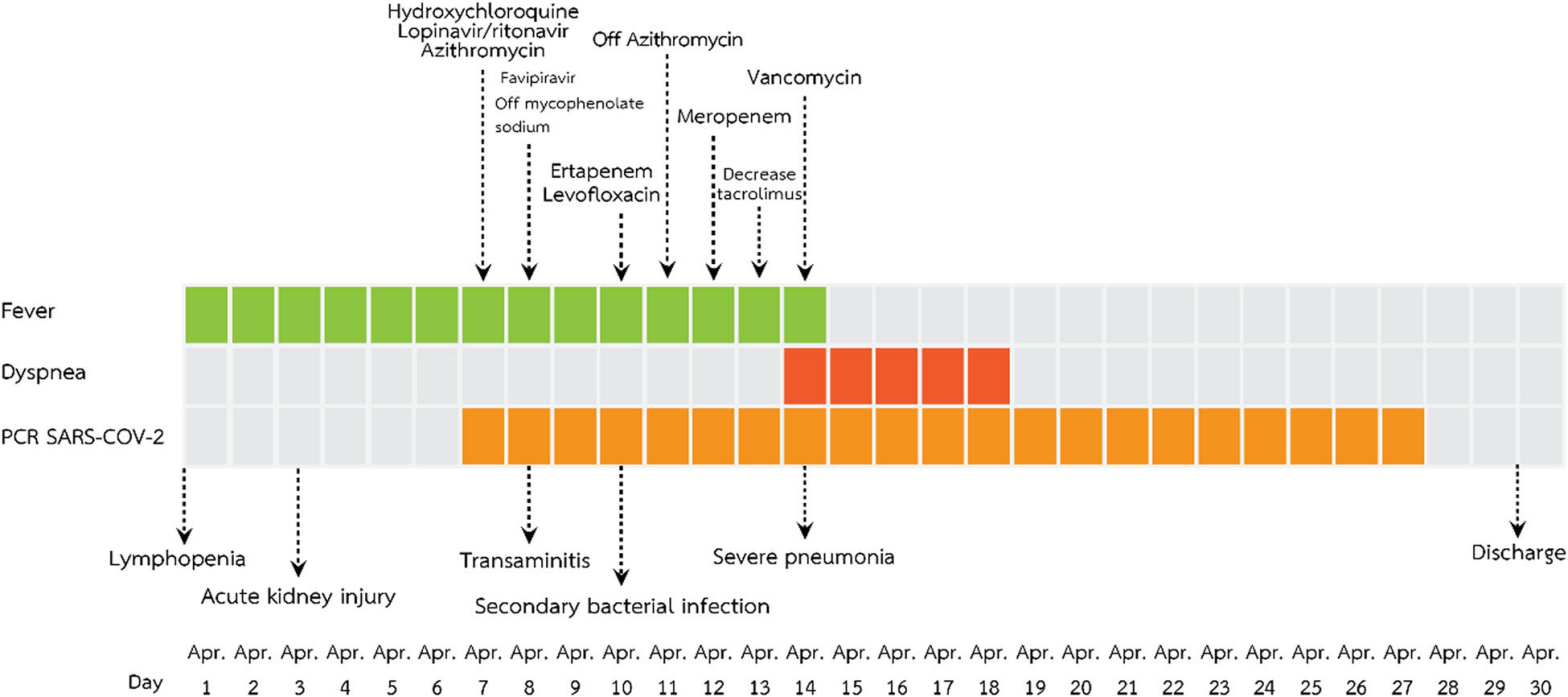
Timeline of the diagnosis and management of a kidney transplant recipient diagnosed with severe COVID‐19 pneumonia (Case 4). COVID‐19, coronavirus disease 2019; PCR, polymerase chain reaction; SARS‐CoV‐2, severe acute respiratory syndrome coronavirus 2

A 38‐year‐old man (a son of Case 1) who underwent a living‐related KT in 2014 who complained of fever and myalgia for 3 days before this admission. He had a history of travel to Bangkok for a follow‐up visit 1 week before this event. His allograft has been maintained on tacrolimus 2 mg/d, mycophenolate sodium 720 mg/d, and prednisolone 5 mg/d. Baseline creatinine and estimated glomerular filtration rate (eGFR) was 0.9 mg/dL and 70.7 mL/minute/1.73 m^2^, respectively. He denied having recently augmented immunosuppression or previous opportunistic infections. He also had chronic hepatitis B on lamivudine 150 mg/d. The exam revealed body temperature of 38.0°C, blood pressure of 100/60 mm Hg, heart rate 60/minute and respiratory rate 16/minute. Otherwise was unremarkable. The initial blood test and CXR were normal. A diagnosis of COVID‐19 was suspected based on a history of contact with a confirmed COVID‐19 case (a primary case). He was empirically treated with oral oseltamivir and oral cefixime while waiting for nasopharyngeal and throat swab for SARS‐Co‐V2 PCR and influenza antigen, which both later revealed negative. However, his fever had persisted until day 6 of admission without localizing symptoms or signs hence for a reassessment for SARS‐CoV‐2 PCR from nasopharyngeal and throat swab, which reported as positive. The CXR also showed small patchy opacities at the left lower lung field, which was compatible with left lower lung COVID‐19 pneumonia. Hydroxychloroquine 800 mg/d, ritonavir‐boosted lopinavir 800 mg/d, azithromycin 250 mg/d, and favipiravir 1200 mg/d were initiated. Mycophenolate sodium was withdrawn, tacrolimus was decreased for low trough level and prednisolone was decreased to 15 mg/d. Intravenous ertapenem and levofloxacin were empirically started 2 days after clinical deteriorating. One week after treatment, he still had persistent fever, tachypnoea as well as new‐onset of diarrhea. The exam revealed respiratory rate of 28/minute with oxygen saturation 95% on oxygen 3 LPM. His labs included WBC 6200 cells/mm^3^, N 88%, creatinine 3.01 mg/dL, lactate dehydrogenase (LDH) 292 IU/mL, CRP 96.1 mg/L, ferritin 2790 ng/mL, and tacrolimus level 66.3 ng/mL. Temporal change in laboratory markers were shown in Figure 5A to C. The repeated CXR revealed a progression to diffuse bilateral infiltrates (Figure 4). Reassessment for nasopharyngeal and throat swab for SARS‐CoV‐2 PCR, which reported as negative, but sputum was reported as positive. His respiratory status has been deteriorating. He was supported by a high‐flow nasal cannula oxygen therapy then practical application of awakening prone ventilation for ARDS. Antibiotic was further escalated to intravenous meropenem for the assumption of secondary bacterial infection. Finally, his clinical and CXR had improved, medications were stopped after 10 days of treatment without adverse reaction. The PCR for SARS‐CoV‐2 from sputum turns to be negative after 20 days of treatment. Except for serum creatinine, which was increased to 2 mg/dL, the rest were decreased, including GFR 40.83 mL/minute/1.73m^2^, CRP 1.9 mf/L, LDH 142 IU/L, and serum ferritin 1459 ng/mL. Timeline after the onset of illness was shown in Figure 6. His hospitalization was complicated by *Clostrioides difficile* colitis requiring oral vancomycin and intravenous metronidazole.

## 3 DISCUSSION

A family cluster of laboratory‐confirmed COVID‐19 cases were reported from the setting where a lockdown policy was implemented. The manifestations were variable ranging from mild upper to severe lower respiratory tract disease leading to ARDS. These occur among family members of different ages and comorbidities. This case series confirmed a transmission among humans of SARS‐CoV‐2 is plausible, as documented in chronological time. Although acute hypoxemic respiratory failure from COVID‐19 in elderly and KT recipients in our cohort seemed to be prominent, early investigation in high‐risk populations, prompt initiation of potential therapy, and intensive supportive care are important to prevent adverse consequences and mortality.

COVID‐19 has been emerging as respiratory tract illness and spreading worldwide. Although the majority could only experience mild respiratory symptoms, those with comorbidities, especially solid organ transplant (SOT) recipients, could potentially develop acute hypoxemic respiratory failure requiring ventilator support likely due to their immunosuppressed conditions.^2^ Although fever and respiratory symptoms are common, atypical presentations such as afebrile, diarrhea, or fatigue without obvious respiratory symptoms were also reported.^19^, ^20^ Chen et al^21^ reported three closed family members, which included a KT recipient husband, his wife, and his son, who live in the same household contracting the disease from unknown sources during an outbreak in China. Our case series could instead identify an index and primary cases, two of them (Cases 1 and 4) contacted with primary case and one of them (Case 3) contacted with an index case as stated in a timeline. Although they did not live in the same household however, in the same neighborhood with frequent visits among each other. So that this finding affirmed a human‐to‐human transmission which explain this communicable disease in a community.^10^ In our cases, we believe that an index case was possibly acquired the virus from direct contact with a primary case (her daughter) who shed the virus even subsided or contaminated with the environment.^22^ This at least confirmed persistent viral shedding after clinical recovery to a previous study which has been reported over a month (37 days) from the onset of symptoms, especially those received delayed treatment.^23^, ^24^, ^25^, ^26^ The information to prove if this is only nonviable shedding or infectious particle is difficult to determine since this will require a viral culture that was not performed and practical in our setting. We determined that our patients had a mean incubation period within 1 week, which was comparable to the previous report.^27^ All patients presented with mild illnesses on admission. Apart from fever and shortness of breath, which were common symptoms in our cohort, muscle ache and dry cough were among the most prevalent presentation reported in the literature.^2^, ^3^, ^4^ Our case series at least supported the illnesses severity variably by immune conditions. The clinical course which was reported included mild disease (81%), severe disease (14%), and critically severe conditions (5%).^5^ Risk factors for severe illness include older age, chronic medical conditions, and immunocompromised conditions such as those receiving immunosuppressive drugs to maintain their transplanted allograft as in our patient.^6^, ^7^, ^8^ RT‐PCR was the useful diagnostic test for COVID‐19 because of short‐turnaround time with high sensitivity and specificity. However, those with immunocompromised conditions as in our KT recipient should be continued to monitor closely for potential progression of the disease even with an initial negative result.^20^ We used two‐step qualitative real‐time RT‐PCR assays to detect two different regions, including the N gene and the Orf1ab gene, which are highly conserved among SARS‐CoV‐2 for early diagnosis. The amplification efficiencies of ORF1b and N gene assays were 99.6% and 95.4%, respectively, in the previous study.^28^

In addition, viral culture and serological diagnosis were not available due to limitations of serological validation and viral culture take times and require biosafety level‐3 for laboratory work.^29^, ^30^ However, KT recipients had negative tests from upper respiratory tract specimens at the first time. Detection of SARS‐CoV‐2 RNA from lower respiratory tract specimens testing are more often positive. Bronchoalveolar lavage fluid showed the highest positive rates 93% followed by sputum 72%, oropharyngeal swab and nasal swab 32% and 63%, respectively.^31^ Comparable with the finding that upper respiratory tract specimens may miss early infection and the main site of viral replication may shift to lower respiratory tract, low infectious burden, or sampling variability. Two cases had severe pneumonia with common patterns of patchy opacities in peripheral and basal both lungs, as previously reported.^32^, ^33^, ^34^ However, we did not routinely perform computed tomography of the chest due to limited accessibility. Therefore, we were unable to definitely differentiate severe pneumonia between COVID‐19 and other organisms since the imaging may be overlapping with other types of pneumonias.^35^

The transplantation society and the Infectious Diseases Society of America recommended confirming COVID‐19 diagnosis in transplant recipients with clinical presentation plus nucleic acid amplification test (NAAT) to detect the RNA of the virus.^36^, ^37^ Furthermore, asymptomatic individuals who are either known or suspected to have been exposed to COVID‐19 should be tested, although with very low certainty of evidence. Our cluster supported an investigation in those with known exposure. We observed that immunosuppressive drugs might obscure or cause atypical presentation early of the illness. The risk of contracting SARS‐CoV‐2 may vary under different exposure conditions from within the same household or staying in the same environment for a certain period of time, especially without wearing appropriate personal protective equipment. In Thailand, a national authority's protocol was encouraged to investigate patients at risk due to a nonuniversal availability of the tests.^37^

Apart from potential antiviral therapy, adjunctive therapies, and respiratory support, adjustment of immunosuppression is another cornerstone which needs to be focused among immunocompromised patients. The Department of Disease Control, Ministry of Public Health, Thailand, recommended hydroxychloroquine and boosted protease inhibitors, either lopinavir or darunavir, were given to those with mild (with comorbidities) and moderate symptoms without pneumonia. Favipiravir was suggested for those diagnosed with pneumonia.^17^ In those with severe COVID‐19 pneumonia, impending respiratory failure, non‐severe pneumonia but with risk factors such as elderly (age of 70 years and older), diabetes mellitus, pulmonary, or cardiac diseases, heavy smoking, body mass index greater than 30 kg/m^2^ and eGFR less than 30 mL/minute/1.73 m^2^, receiving lymphocyte depletion therapy within 3 to 6 months, all immunosuppressants would need to discontinue and increase/start steroids at 15 to 25 mg/d. The exception is to attempt to keep dual therapy CNI‐steroids on those without risk factors. Antiviral agents such as favipiravir and remdesivir have been reported in the literature for potential effectiveness, although few immunocompromised patients were included.^38^, ^39^ So management was composed of immunosuppression reduction and supportive therapy and almost of transplant patients had good outcome and few could succumb were reported.^40^ Therefore, clinicians should be highly aware of, and test for COVID‐19, including re‐evaluation, should be considered in immunocompromised populations even though they had an atypical presentation or initially negative results.^20^ Therefore, further studies focused on immunocompromised patients are needed to be followed.

The European Renal Association‐European Dialysis and Transplant Association recommended all immunosuppressants to be continued in patients without symptoms. On the other hand, discontinuation of antimetabolites such as mycophenolic acid or azathioprine, and mammalian target of rapamycin (mTOR) inhibitors and maintained on a lower therapeutic range of calcineurin inhibitors (CNIs); 2 to 4 ng/mL for tacrolimus and 35 to 65 ng/mL for cyclosporin; and low‐dose steroid is recommended for symptomatic patients without pneumonia. Instead, all immunosuppressants would need to be discontinued among those with pneumonia.^41^

Drug interactions could possibly increase CNI and mTOR inhibitors while concurrent use with hydroxychloroquine. Therapeutic monitoring of their trough levels should be considered.^42^ Furthermore, CNIs and mTOR inhibitors must be stopped in those starting ritonavir‐boosted protease inhibitors. In our case, the trough level of tacrolimus was supratherapeutic due to the effect of cytochrome inhibition of ritonavir to tacrolimus and decreased in allograft function, although a sole effect could not be distinguished. Another aspect is when to resume the immunosuppressant to maintain an allograft is still elucidated and needed to be individualized based on immunologic risk of rejection in each patient. We decided to resume half a dose of mycophenolic acid 72 hours after being afebrile and respiratory stable.

Sepsis, ARDS, and multiorgan failure have been reported as complications from COVID‐19. The onset of pneumonia, ARDS, and superinfection in two cases were in the second weeks, which were comparable to the previous study.^2^ Additionally, we found that severe lymphocytopenia, elevated ferritin, and CRP were correlated with disease severity, which was affirmed in our transplant recipient.^2^, ^8^, ^43^ Three out of four patients had progression to pneumonia. Clinical spectrum of pneumonia from mild pneumonia to ARDS. Although bacterial or fungal superinfection post‐respiratory infection has been reported, we instead did not recover a pathogen in this caveat although antibiotic was escalated for this possible concern.^44^ Therefore, older age, male sex, underlying conditions, and laboratory markers including lymphopenia, high CRP, D‐dimer, ferritin, and high‐sensitivity cardiac troponin I levels are essential to predict the severity and mortality of COVID‐19.^19^, ^40^, ^45^, ^46^ Meta‐analysis showed that lymphocytopenia was associated with severe COVID‐19.^46^ The majority of COVID‐19 patients in the general population have leukopenia with lymphocytopenia (70%) as similar to transplant recipients.^47^, ^48^ In our cases, severe lymphocytopenia was found in transplant recipients when markedly deteriorated to severe pneumonia. This is comparable with lymphocyte sequestration in target organ due to acute SARS‐CoV‐2 infection and effect of the immunosuppressive drug. Additionally, acute kidney injury in the fourth patient was most likely due to an impact of CNI on renal hemodynamic and tubular dysfunction^49^; other causes were hypovolemia from high‐grade fever and diarrhea, sepsis‐induced, impairment of gas exchange due to severe pneumonia, and microcirculatory derangement from inflammatory reaction.^50^ Few KT recipients who were infected with SARS‐CoV‐2 have been reported in the literature.^18^, ^51^, ^52^ Compare to our patient, the clinical presentation of COVID‐19 in SOT recipients, especially KT recipients, have been reported sporadically during a current outbreak. A large cohort to better conclude a harmonization of the patients and few immunocompromised patients included still limited to be concluded the guideline for our immunocompromised patients.

Finally, all patients had a clinical response to the treatment and negative PCR for COVID‐19. Repeated testing was done for all cases, and persistent positive RT‐PCR from lower respiratory tract specimens was found in KT recipient who had severe pneumonia. We assumed that sustained viral shedding beyond 14 days had been reported in several respiratory viral infections in immunocompromised patients such as influenza and lower respiratory tract specimens have high yield for late detection and monitoring of patients with severe COVID‐19 pneumonia.^30^, ^53^ However, tested clinical samples were done from different sites and onset times. In addition, these tested qualitatively; the exact viral copy numbers cannot be determined. So it is premature to determine the viral replication kinetics.^28^ The rate of mortality has been reported ranging from as low as 2% to 10% depends on health care capacities and medical supplies.^20^, ^42^, ^50^, ^54^ Our patients were middle‐aged to the elderly. Two are elderly with no medical condition and COPD each. Another two young patients carried no comorbidity and KT. However, there was no mortality in our cohort likely due to early diagnosis and management in our patients.

Our case series had a few limitations. First, the underestimation in the correlation between laboratory value and disease severity could be inevitable due to lacking all blood tests in all patients, especially those with mild disease because the results would not deem to influence the management. Second, the duration of viral shedding is limited due to limited NAAT testing in our setting. Third, we were not able to provide solid evidence of person‐to‐person transmission since we did not genetically investigate a whole‐genome sequencing of the virus. Last, a sole effect of favipiravir in our cases could not be concluded due to a confounding effect of multiple adjunctive therapies along with an adjustment of immunosuppressant. However, our case at least supports early multifacet management, including antiviral therapy, adjunctive therapy, adjustment of immunosuppressant and intensive care could prevent morbidity and mortality in these specific immunocompromised hosts.

In summary, a family cluster of COVID‐19 had varied clinical presentation from mild infection to severe pneumonia complicated with ARDS. The main management is comprised of investigational antiviral therapy, adjustment of immunosuppressant, and respiratory support. Clinicians who are encountering transplant recipients should be aware of a possibility of transmission among the family members and the severe course of the disease.

## CONFLICT OF INTERESTS

The authors declare that there are no conflict of interests.

## AUTHOR CONTRIBUTIONS

PS, JB, CP, and NC: conceptual designing of the study; PS and JB: collecting data and drafting the manuscript. All authors have reviewed and approved the manuscript submission.

## ETHICS STATEMENT

This study is approved by the medical ethical review of Lampang Hospital, Thailand. Subjects were informed of the purposes of the study and gave their signed informed consent.

## Data Availability

The data that support the findings of this study are available on request from the corresponding author. The data are not publicly available due to privacy or ethical restrictions.
